# Ethical perceptions of DRG-based hospital financing among physicians and hospital managers in Germany: a cross-sectional survey

**DOI:** 10.1186/s12910-026-01498-0

**Published:** 2026-06-03

**Authors:** Sara Becker, Henning Lemm, Andreas Stang, Samuel Sossalla, Michael Buerke, Priyanka Boettger

**Affiliations:** 1 Department of Internal Medicine III, Cardiology Martin Luther University Halle (Saale), Halle (Saale), Germany; 2 Medical Clinic II, Heart and Vascular Center South Westphalia, St. Marien Hospital Siegen, Siegen, Germany; 3 Center for Clinical Epidemiology, IMIBE, University Hospital Essen, Essen, Germany; 4 Department of Health Policy, London School of Economics and Political Sciences, London, UK; 5 Department of Internal Medicine I, Cardiology, Angiology and Critical Care Medicine, University of Giessen, Klinikstrasse 33, Giessen, 35392 Germany

**Keywords:** DRG system, Medical ethics, Hospital reform, Health economics, Professional identity, Moral distress, Patient care

## Abstract

**Background:**

Diagnosis-Related Groups (DRGs) have reshaped German hospital financing by linking reimbursement to case-based payment. While intended to increase efficiency, DRG-based reimbursement has been associated with concerns regarding professional autonomy, patient-centeredness, and organizational ethics. This study examines how physicians and hospital managerial stakeholders perceive the DRG system, focusing on professional identity, patient care, and moral conflict.

**Methods:**

We conducted a cross-sectional online survey among hospital physicians and managerial stakeholders in two German federal states (North Rhine–Westphalia and Saxony-Anhalt). The questionnaire assessed perceived economic pressure, ethical conflict, and quality-of-care implications under DRG conditions. Descriptive comparisons and multivariable logistic regression were used to estimate associations between professional role and reported ethical concerns.

**Results:**

Physicians more frequently reported perceived moral conflict (82% vs. 34%), reduced patient-centeredness (76% vs. 39%), and time pressure associated with DRG-linked hospital financing (85% vs. 71%) than managerial respondents. Managerial stakeholders more frequently emphasized efficiency and coordination gains. Both groups supported the use of quality indicators to protect care standards, though physicians were more likely to call for rebalancing financial and clinical priorities.

**Conclusions:**

Physicians and managerial stakeholders perceive DRG-linked incentives differently, and both groups identify ethical pressure points in current hospital financing. The findings suggest that hospital financing reforms should consider not only efficiency incentives but also their perceived interaction with professional ethics, continuity of care, and organizational culture.

**Supplementary Information:**

The online version contains supplementary material available at 10.1186/s12910-026-01498-0.

## Introduction

Until the early 2000s, German hospitals were reimbursed primarily through per diem rates and department-based budgets. Between 2003 and 2004, this system was replaced nationwide by the German diagnosis-related group (G-DRG) system, which introduced fixed case-based payments for inpatient treatment. Designed to improve efficiency and cost control in inpatient care, the DRG system created incentives to reduce length of stay and increase case turnover, thereby contributing to a more economically driven hospital environment [1]. While proponents emphasize improvements in coordination and financial transparency, concerns have emerged regarding mounting economic pressure and changes in medical decision-making. Although DRG systems exist internationally, important structural differences limit direct comparability. The German G-DRG system combines nationwide standardized reimbursement categories with corporatist hospital financing structures and predominantly salaried physicians. Consequently, international findings regarding gaming behavior, patient selection, or physician incentives require cautious interpretation in the German context.

Hospitals increasingly operate at the intersection of partially competing institutional logics. Clinical logic prioritizes individualized patient welfare, professional autonomy, and continuity of care, whereas managerial logic emphasizes efficiency, accountability, resource stewardship, and organizational sustainability. Case-based reimbursement systems such as DRGs may intensify these tensions because financial performance becomes more closely linked to throughput, coding structures, and length-of-stay management. Previous studies from Germany, Switzerland, and other DRG-based systems suggest that physicians and hospital managers may therefore perceive economic incentives differently depending on their professional responsibilities and ethical orientation. This distinction can also be interpreted through sociological theories of professionalism and institutional logics, in which professional medical logic emphasizes fiduciary responsibility, patient advocacy, and professional autonomy, whereas managerial logic prioritizes efficiency, organizational performance, and economic accountability.

In particular, physicians face increasing difficulty reconciling professional and ethical duties with institutional efficiency targets. Rooted in the principle of “primum non nocere” (first, do no harm), the medical ethos has traditionally prioritized individual patient welfare above financial considerations [2]. Under DRG conditions, however, clinicians and hospital managers are expected to align treatment decisions with hospital budgets and throughput goals, generating ethical tension and perceived role conflict [3]. International literature identifies several core ethical concerns associated with DRG-based reimbursement. These include risks to equity and access to care, particularly for patients with multimorbidity or social vulnerability [4–6]; concerns regarding quality and safety of care due to pressure for early discharge and service limitation [5, 7]; erosion of professional autonomy and increasing reliance on administrative performance indicators [4]; as well as strategic behaviors such as upcoding, fragmented treatment, and avoidance of high-risk cases [5]. Additional concerns include reduced transparency and accountability caused by complex DRG algorithms and data manipulation [8], alongside potential disadvantages for vulnerable patient groups whose care needs are insufficiently reflected within reimbursement structures [6].

In light of these concerns, ethicists and health policy experts have called for systematic evaluation of DRG impacts across multiple domains. The ethical implications of DRG-based financing can be interpreted through complementary frameworks including principlism, distributive justice, professional ethics, and moral distress theory. These frameworks emphasize that financing systems should preserve equitable access, continuity of care, and professional integrity in addition to economic efficiency [2, 6].

Against this backdrop, the present study explores how physicians and managerial stakeholders in Germany perceive the DRG system, focusing on professional identity, patient care, and moral distress. Drawing on a cross-sectional survey conducted in two federal states, the analysis compares clinical and managerial perspectives on the ethical and operational implications of DRG-driven hospital management. In the context of current reforms aimed at correcting financial incentives and introducing standby financing (“Vorhaltefinanzierung”) [9], the study provides empirical insight into the ethical dimensions of contemporary hospital financing. Based on theories of institutional logics and professionalism, we assumed that physicians and managerial stakeholders would perceive DRG-linked incentives differently because of their distinct professional responsibilities and organizational roles. Physicians are directly involved in bedside decision-making, continuity of care, and patient advocacy, whereas managerial stakeholders are more strongly engaged in budgeting, organizational governance, and resource allocation. We additionally explored whether perceptions differed according to hospital ownership and regional context, as organizational structures, financial pressures, and implementation environments may vary between public, charitable, and private hospitals as well as between western and eastern German federal states.

We hypothesized that physicians would report higher levels of perceived moral conflict and reduced patient-centeredness than managerial stakeholders because physicians are more directly involved in bedside decision-making. We additionally explored whether perceptions differed according to hospital ownership and regional context.

## Methods

This study employed a cross-sectional survey design to examine perceptions of the DRG system among physicians and hospital managerial stakeholders in Germany. The online survey was conducted in two federal states: North Rhine-Westphalia and Saxony-Anhalt (Supplement S3). North Rhine-Westphalia and Saxony-Anhalt were selected because they differ in hospital density, ownership structure, and regional healthcare organization, thereby allowing comparison between a large western federal state and a smaller eastern federal state with different structural conditions. The survey invitation was distributed to 275 hospitals (238 from North Rhine-Westphalia and 37 from Saxony-Anhalt), of which 2/3 agreed to participate. A total of 3662 invitations were sent, yielding 529 completed questionnaires and a response rate of 19%. The survey was conducted over 6 month period with several reminders at 2020. Respondents were not asked to retrospectively compare current practice with the pre-DRG era. Instead, survey items assessed current perceptions of how DRG-linked incentives influence clinical and managerial practice in contemporary hospitals. The target population included senior hospital physicians and administrative staff from public, charitable, and private hospital providers. Specifically, each hospital was asked to nominate its chief physician and at least one senior consultant from departments of internal medicine (including cardiology, gastroenterology, oncology), general surgery, orthopedics, and gynecology. Additionally, administrative leaders such as managing directors, heads of controlling, and human resources managers were invited to participate. Chief physicians and senior consultants were included because they combine clinical responsibility with managerial tasks, whereas hospital managerial stakeholders perform primarily managerial functions. German hospital physicians are salaried employees. Chief physicians may receive supplementary income from private patients, but remuneration is generally not directly linked to DRG-related performance indicators. Administrative respondents were included because they occupied positions involving budgeting, controlling, staffing, or organizational governance and therefore contributed to the institutional incentive structures within which clinical decisions are made.

**Fig. 1:**
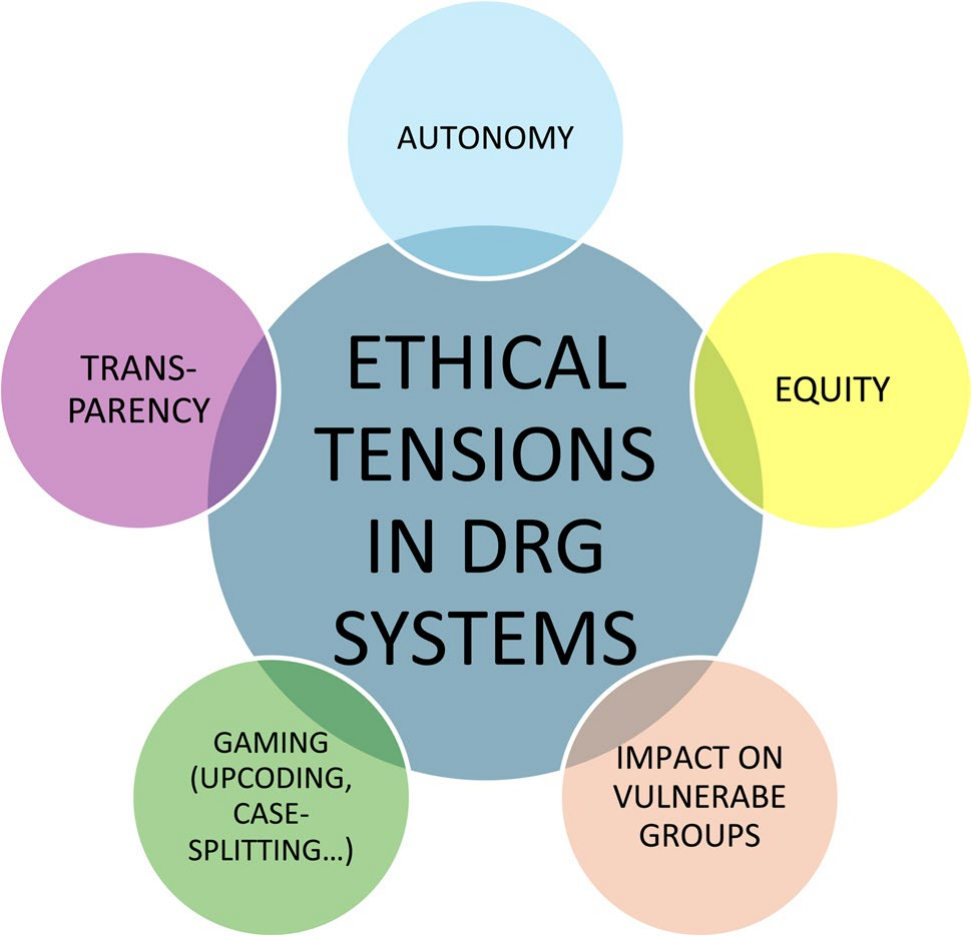
Ethical Tensions in DRG Systems: A Conceptual Framework This conceptual diagram illustrates six core ethical tensions associated with Diagnosis-Related Group (DRG) systems in hospital financing. These include: Equity – risks of unequal access and patient selection; Autonomy – constrained clinical decision-making; Transparency – limited accountability and complex coding; Gaming – strategic manipulation (e.g. upcoding, case-splitting); Vulnerable Groups – potential disadvantage to complex or underserved patients; Quality of Care (implicitly addressed through systemic consequences across all domains)

Questionnaire domains were derived from prior literature on DRG-related ethical tensions, professional role conflict, and moral distress, including the ethical framework proposed by Fourie et al. Draft items underwent iterative review by clinicians, hospital managers, and health policy researchers to establish face validity and conceptual clarity. A pilot version was tested among five senior physicians and three managerial respondents, leading to minor revisions in wording and item sequencing. The standardized questionnaire consisted predominantly of closed-ended items and was structured around five thematic modules Fig. 1:

- Economic orientation and professional identity (e.g., self-conception as physician vs. manager).
- The role of economics in hospital care (e.g., perception of financial incentives and profit orientation).
- Patient care conditions (e.g., work environment, time for communication, treatment quality).
- Hospitals as market actors (e.g., changes perceived since DRG implementation).
- Ethics and values (e.g., moral conflict between economic imperatives and clinical ethos).

Responses to the main questionnaire items were collected using a six-point Likert scale ranging from 1 = strongly agree to 6 = strongly disagree. A six-point scale was chosen deliberately to avoid a neutral midpoint and encourage respondents to indicate the direction of their opinion. For analysis, responses were dichotomized into agreement (1–3) and disagreement (4–6). Self-assessed familiarity with DRG mechanisms was collected separately on a 10-point scale and was not part of the main questionnaire. The full questionnaire used in this study is provided in Supplementary Material S3. Direct DRG experience was defined as active involvement in coding, case review, departmental budgeting, controlling, hospital management, or DRG-related administrative decisions.

### Data handling, missing data, and statistical analysis

Participation was voluntary and anonymous. Because invitations were distributed through hospital leadership to senior physicians and administrative staff, self-selection cannot be excluded; the overall response rate was approximately 19%. Item-level missingness for key variables was low (< 5%). Missing responses were coded as non-response and excluded using pairwise deletion for descriptive analyses and complete-case analysis for regression models. No post-stratification weighting was applied. Responses to the main questionnaire items were collected using a six-point Likert scale and dichotomized into agreement (scores 1–3) and disagreement (scores 4–6) for analysis. Because Likert responses represent ordinal data, primary analyses focused on categorical comparisons and dichotomized agreement categories rather than assuming interval-level measurement properties. Descriptive statistics were used to summarize demographic characteristics, professional roles, and response distributions across survey items. Group differences between physicians and managerial stakeholders were examined using chi-square tests for categorical variables and t-tests or Mann–Whitney U tests for continuous variables, depending on the distribution. Statistical significance was defined as *p* < 0.05 (two-sided). Based on the conceptual framework outlined in the Introduction and prior literature on institutional logics, professional role conflict, and organizational variation in DRG implementation, binary logistic regression models were constructed to explore factors associated with perceived ethical tensions, including moral conflict, reduced patient-centeredness, and economic influence on medical decisions.

Covariates included years of professional experience, hospital ownership, hospital level (basic, specialized, university), and region (North Rhine-Westphalia versus Saxony-Anhalt). Effect estimates are reported as odds ratios (ORs) with 95% confidence intervals (CIs), and interaction terms were tested where conceptually relevant (e.g., profession × ownership type). Respondents with missing professional-role information were retained for overall descriptive analyses but excluded from profession-specific subgroup analyses.

All analyses were performed using R version 4.3.2 (R Foundation for Statistical Computing, Vienna, Austria), SPSS version 29.0 (IBM Corp., Armonk, NY), and Stata version 18.0 (StataCorp LLC, College Station, TX).

## Results

### Sample characteristics

**Fig. 2:**
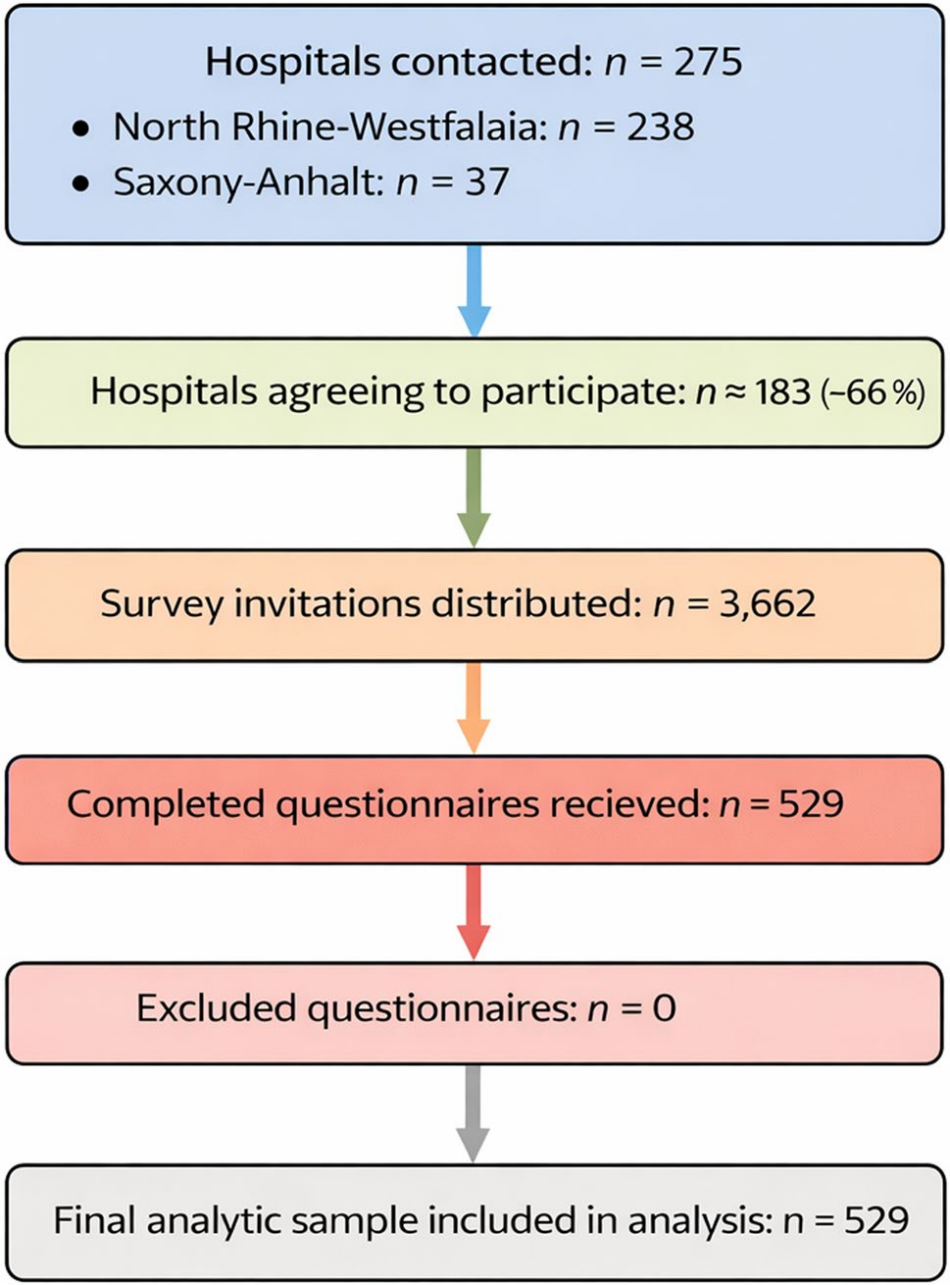
STROBE-style participant flow diagram. The survey invitation was distributed to 275 hospitals (238 in North Rhine-Westphalia and 37 in Saxony-Anhalt), of which approximately two-thirds agreed to participate. A total of 3,662 invitations were distributed, yielding 529 completed questionnaires (response rate 19%). No questionnaires were excluded; therefore, all 529 responses were included in the final analysis

A total of 529 professionals participated in the survey, corresponding to a response rate of approximately 19%. Figure 2 presents the participant flow. The survey invitation was distributed to 275 hospitals (238 in North Rhine-Westphalia and 37 in Saxony-Anhalt), of which approximately two-thirds agreed to participate. A total of 3,662 invitations were sent, yielding 529 completed questionnaires.

Of these, 98 were women (19%) and 431 were men (81%). The predominance of male respondents reflects the gender distribution of senior hospital physicians and managerial stakeholders in Germany, where leadership positions remain occupied predominantly by men. The majority of participants were physicians: 175 (33.1%) were department heads (chief physicians), and 301 (56.9%) held senior physician positions. A smaller proportion represented administrative roles: 18 (3.4%) from hospital management, 17 (3.3%) from controlling, and 5 (1.0%) from human resources. For 13 respondents (2,4%), professional-role information was missing; these respondents were retained in the overall descriptive sample but excluded from profession-specific subgroup analyses. Most respondents (95%) had more than 10 years of professional experience. With regard to institutional setting, 49% worked at primary or secondary care hospitals, 27% at specialized care hospitals, and 24% at university hospitals. In terms of ownership structure, 39% were employed in publicly owned institutions, 48% in non-profit (charitable) organizations, and 13% in private for-profit hospitals.

This diverse sample allows for meaningful comparisons between medical and administrative stakeholders regarding their perspectives on the DRG system. The average self-reported familiarity with DRG mechanisms was 7.1/10 (SD 1.9) using a separate 10-point self-rating scale. The survey revealed substantial differences between physicians and hospital managerial stakeholders in their perceptions of the DRG system’s impact on professional roles, patient care, and ethical practice. The results are structured according to the core thematic domains of the questionnaire. The characteristics of the respondents and their degree of experience with the DRG system are summarized in Fig. 2; Table 1.

**Table 1:** Respondent characteristics (sex, profession, years of experience, hospital type, ownership, direct DRG experience)

Characteristic	*n*	%
Sex		
Women	98	19.0
Men	431	81.0
Professional role		
Chief physicians / department heads	175	33.1
Senior physicians / consultants	301	56,9
Hospital management	18	3.4
Controlling	17	3.2
Human resources	5	1
Professional role missing	13	2,4
Years of professional experience		
> 10 years	503	95.0
≤ 10 years	26	5.0
Hospital type		
Primary/secondary care hospital	259	49.0
Specialized care hospital	143	27.0
University hospital	127	24.0
Ownership		
Public	206	39.0
Non-profit / charitable	254	48.0
Private for-profit	69	13.0
Direct DRG experience		
Yes	413	78.0
No	116	22.0

Direct DRG experience was defined as active involvement in coding, case review, departmental budgeting, hospital controlling, management, or DRG-related administrative decision-making. The predominance of male respondents reflects the current gender distribution of senior hospital physicians and managerial stakeholders in Germany. Data derived from the study sample (*n* = 529).

### Professional identity and economic orientation

A substantially greater proportion of physicians than managerial stakeholders perceived a clear distinction between medical and managerial professional identities (84% vs. 55%, *p* < 0.001), suggesting that clinicians more often experienced tension between their traditional professional role and the managerial expectations associated with DRG-based hospital financing.

Despite this, a majority of physicians (85%) stated they assumed managerial responsibilities, and 82% acknowledged that economic considerations influenced medical decision-making. All managerial stakeholders (100%) believed that senior physicians should adopt a managerial mindset, reflecting a strong institutional expectation (*p* < 0.001). Physicians, in contrast, showed greater ambivalence, with many reporting internal tension between patient advocacy and resource control.

### Predictors of perceived ethical tensions

Multivariable logistic regression models were used to identify factors associated with ethical concerns in DRG-based hospital care (Table 2). Being a physician was a strong and statistically significant predictor of reporting moral conflict due to the DRG system (OR: 2.75; 95% CI: 1.60–4.72; *p* = 0.001) and perceiving reduced patient-centeredness (OR: 1.89; 95% CI: 1.12–3.19; *p* = 0.018), compared to administrative staff. In addition, private hospital ownership was associated with higher odds of perceiving patient-centeredness as compromised (OR: 1.48; 95% CI: 1.02–2.13; *p* = 0.039). Respondents from North Rhine–Westphalia (NRW), compared with those from Saxony-Anhalt, were more likely to report fragmentation of care, although this association did not reach statistical significance (OR = 1.36; 95% CI 0.95–1.93; *p* = 0.092). These findings suggest that regional structural factors, in addition to professional role, shape ethical perceptions of DRG-related hospital care. Furthermore, respondents from private for-profit hospitals more frequently reported perceived economic pressure and reduced patient-centeredness than respondents from public or charitable hospitals. Although effect sizes were modest, this pattern suggests that organizational context may shape how DRG-linked incentives are experienced.

**Table 2:** Predictors of perceived ethical tensions

Outcome	Predictor	Odds Ratio (OR)	95% CI	*p*-value
Moral conflict due to DRG system	Profession: Physician (ref = Admin)	2.75	1.60–4.72	**0.001**
Reduced patient-centredness	Ownership: Private vs. Public	1.48	1.02–2.13	**0.039**
Perceived fragmentation of care	Federal state: NRW (ref = Saxony-Anhalt)	1.36	0.95–1.93	0.092
Support for quality indicators	Profession: Physician (ref = Admin)	1.89	1.12–3.19	**0.018**

All models adjusted for years of experience, hospital level (primary/specialized/university), and ownership (public/non-profit/private)

*OR* odds ratio, *CI* confidence interval, *DRG* Diagnosis-Related Group

Bold *p*-values indicates significance

### Impact on patient care

Physicians were significantly more likely than managerial stakeholders to report that time pressures had increased under the DRG system (85% vs. 71%, *p* = 0.02). A decline in empathy and attentiveness toward patients due to economic pressure was reported by 75% of physicians compared to 43% of managerial stakeholders (*p* < 0.001). Perceived negative effects on care continuity were also more common among physicians. The “revolving door” effect—premature discharge followed by readmission—was reported by 57% of physicians but only 29% of managerial stakeholders (*p* < 0.01). Fragmentation of care episodes for billing reasons (e.g. staged admissions) was noted by 85% of physicians versus 54% of managerial stakeholders (*p* < 0.001).

**Fig. 3:**
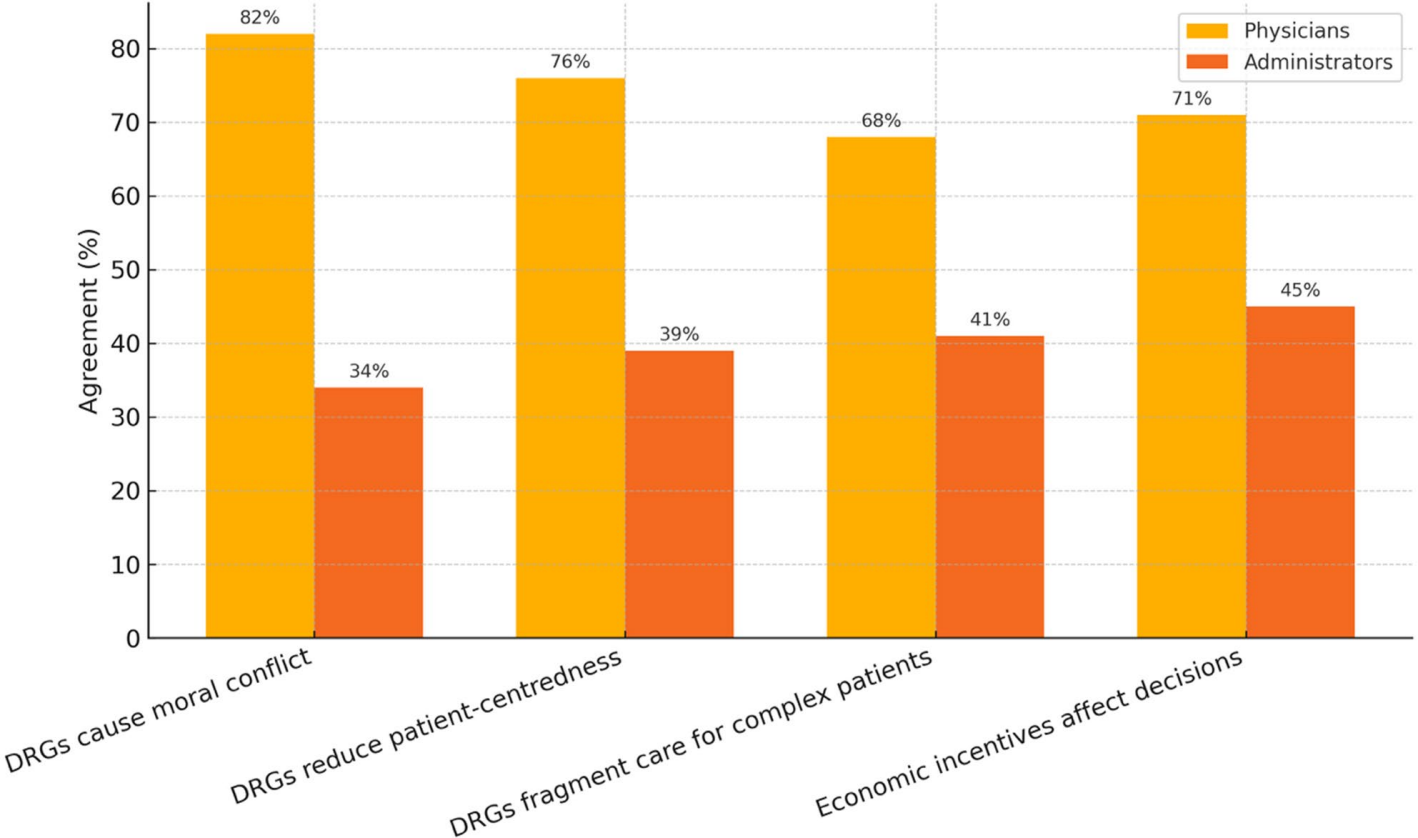
Perceived ethical tensions related to the DRG system among physicians and managerial stakeholders. Responses are shown as percentages of agreement to selected statements reflecting moral conflict, patient-centeredness, and care fragmentation. Physicians were significantly more likely than managerial stakeholders to perceive ethical problems related to DRG incentives, particularly regarding moral conflict (82% vs. 34%) and patient-centeredness (76% vs. 39%). Agreement = proportion of respondents who agreed or strongly agreed with each statement. DRG = Diagnosis-related groups

Furthermore, physicians were significantly less likely to rate overall care coordination and patient-centredness positively (*p* < 0.01 for both), suggesting a more critical view of how DRG logic affects clinical processes Fig. 3.

### Perceptions of economic influence and ethical conflict

Most respondents in both groups recognized an increasing market orientation in hospital governance. A large proportion of physicians (89%) and a substantial majority of managerial stakeholders (69%) agreed that hospitals had shifted from caregiving institutions to economically driven enterprises (*p* < 0.01). Most physician respondents reported perceiving tension between economic constraints and professional obligations (85% vs. 54%, *p* < 0.001).

**Fig. 4:**
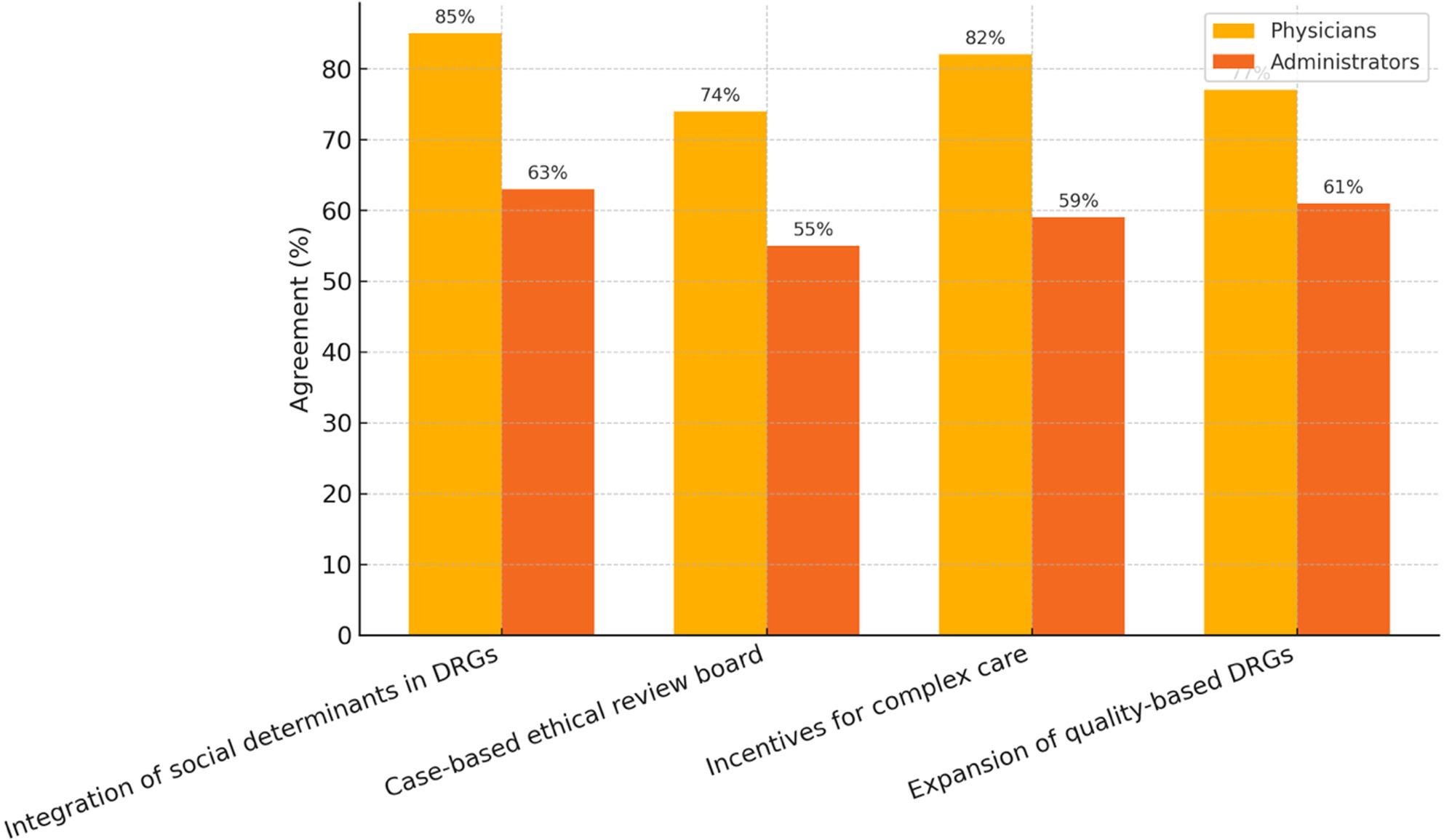
Support for reform proposals to improve ethical performance of DRG systems. The figure shows the percentage of physicians and managerial stakeholders who supported selected reform options. Physicians expressed particularly high support for integrating social determinants into algorithms (85%) and incentivizing complex care (82%). Agreement refers to the proportion rating the proposal as ‘likely important’ ‘important’ or ‘very important’ on a separate six-point scale. Across all proposals, physicians reported higher approval rates than managerial stakeholders, indicating differing ethical priorities and reform expectations between professional groups. Agreement = percentage of respondents who rated the measure as “important” or “very important”. DRG = Diagnosis-related groups

When asked whether hospital care had deteriorated under DRG conditions, 70% of physicians answered affirmatively, compared to 42% of managerial stakeholders (*p* < 0.001). Regular moral conflict was reported more frequently among less senior physician respondents and less frequently among chief physicians (p for trend < 0.01). Chief physicians reported such conflicts least often (*p* < 0.01) Fig. 4.

The figure shows the percentage of physicians and managerial stakeholders who supported selected reform options. Physicians expressed particularly high support for integrating social determinants into algorithms (85%) and incentivizing complex care (82%). Agreement refers to the proportion rating the proposal as ‘likely important’ ‘important’ or ‘very important’ on a separate six-point scale. Across all proposals, physicians reported higher approval rates than managerial stakeholders, indicating differing ethical priorities and reform expectations between professional groups. Agreement = percentage of respondents who rated the measure as “important” or “very important”. DRG = Diagnosis-related groups.

### Attitudes toward performance metrics and hospital mission

Support for quality indicators (e.g. treatment outcomes, readmission rates) was high in both groups—73% of physicians and 100% of managerial stakeholders endorsed their use to safeguard care quality (*p* < 0.001). However, endorsement of economic key performance indicators (e.g. cost-efficiency metrics) was significantly lower among physicians (53%) compared to managerial stakeholders (97%) (*p* < 0.001).

Finally, 91% of physicians and 73% of managerial stakeholders agreed that nonprofit medical care should be strengthened in German hospitals (*p* < 0.01), reflecting broad concern about commercialization and a shared normative orientation—albeit with differing intensity.

### Stakeholder perspectives on ethical tensions

**Fig. 5:**
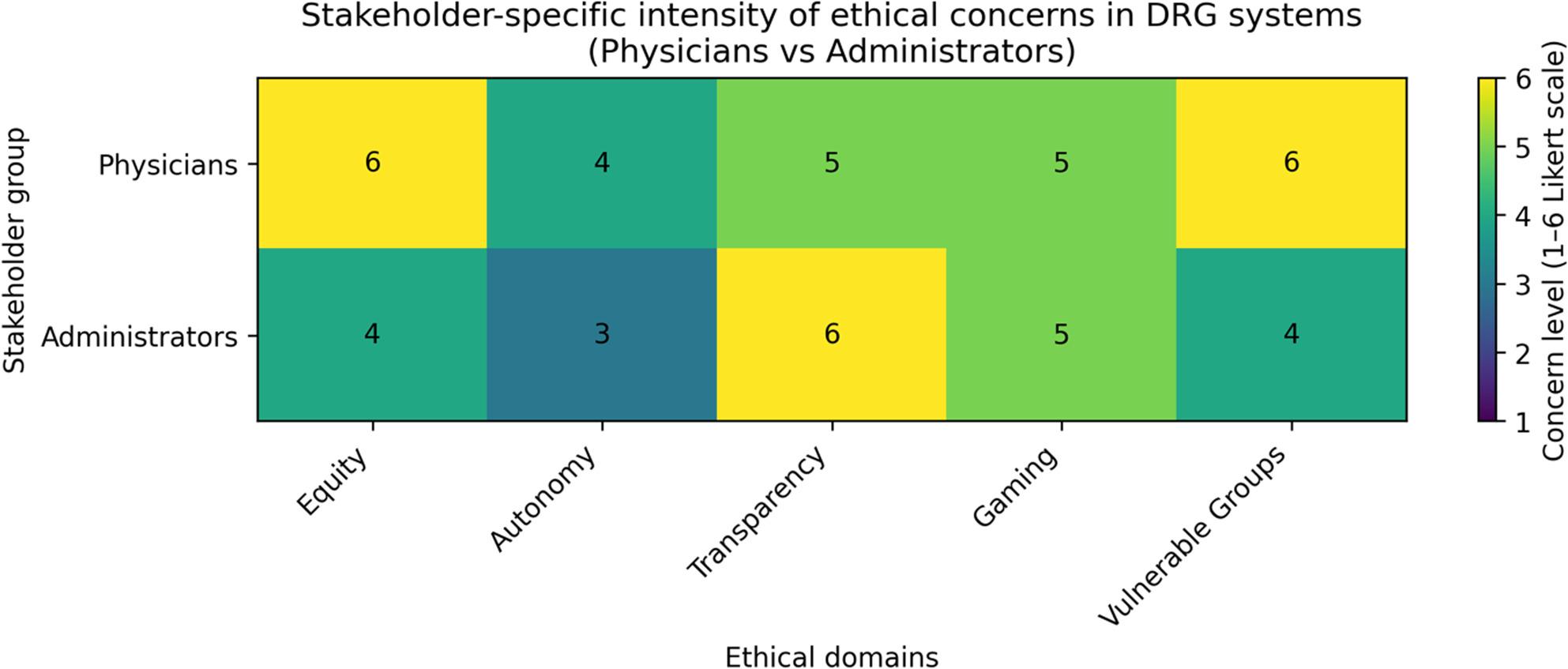
Stakeholder-specific intensity of ethical concerns in DRG systems. Heatmap based on mean agreement scores derived from six-point Likert items (1 = strongly agree, 6 = strongly disagree) across five ethical tension domains, as rated by physicians and managerial stakeholders. Domains include Equity, Autonomy, Transparency, Gaming, and Vulnerable Groups. Physicians rated equity and vulnerable groups highest, while managerial stakeholders expressed peak concern about transparency and systemic gaming. Data derived from stakeholder survey (*n* = 120), Likert-scaled

Stakeholder perspectives on the ethical implications of the DRG system revealed distinct yet convergent patterns of concern (Fig. 5). Physicians expressed the strongest reservations regarding equity and the disproportionate impact on vulnerable patient groups, assigning the highest concern levels to these domains. Managerial stakeholders, by contrast, were primarily focused on transparency, systemic efficiency, and the risk of strategic behavior within reimbursement structures. The consistent identification of “gaming” as a moderate-to-high concern across both stakeholder groups underscores a shared awareness of perverse incentives inherent in the DRG framework. These findings illustrate the dual ethical perspective that characterizes DRG implementation—clinically rooted apprehension about patient equity versus administratively centered concerns about governance and transparency—and point to the necessity of targeted, stakeholder-specific reform strategies.

## Discussion

This study provides empirical evidence on how hospital professionals perceive the ethical implications of DRG-based financing in Germany. The findings describe perceived associations rather than causal relationships. Physicians reported substantially higher levels of moral distress, perceived reductions in patient-centeredness, and greater concern about fragmented care than managerial stakeholders. These differences likely reflect competing institutional logics within hospitals: a clinical orientation centered on individual patient welfare and a managerial orientation focused on stewardship of finite resources, including hospital beds, staffing, operating room capacity, and departmental budgets. These perceptions are consistent with international literature suggesting that cost-driven delivery models may erode professional integrity and contribute to moral distress among clinicians [10–12].

Managerial stakeholders, in contrast, more frequently framed DRGs as a necessary instrument for economic steering and hospital governance [13, 14]. This divergence suggests a persistent gap between clinical and administrative role expectations. Clinicians prioritize obligations toward individual patients, whereas managerial stakeholders emphasize organizational sustainability and efficient resource allocation. Although this pattern is not unique to Germany, our findings indicate that DRG-linked financing may reinforce perceived distance between clinical and managerial priorities. Importantly, these findings should not be interpreted as evidence that DRG-based reimbursement is inherently unethical or fundamentally incompatible with high-quality patient care. Rather, the data suggest that ethical tension emerges when activity-based reimbursement is insufficiently balanced by continuity-oriented quality safeguards, structural support for complex care, and organizational conditions that protect professional autonomy and patient-centeredness.

Despite these differing emphases, both groups identified similar structural pressures: incentives toward shorter length of stay, accelerated discharge, and reduced continuity of care, particularly for patients with multimorbidity or complex psychosocial needs [15]. These observations align with previous reports from DRG-based systems describing early discharge, patient selection, and strategic coding behavior [5, 16]. The extent to which such pressures are experienced as ethically problematic may also depend on local leadership practices and communication of economic targets.

A notable finding was the strong physician support for quality indicators, especially measures reflecting continuity of care, complication avoidance, and sustained responsibility for vulnerable patients. This suggests that clinicians are not opposed to accountability or measurement itself, but rather to performance regimes perceived as prioritizing throughput over quality. Participants frequently perceived DRG-linked incentives as favoring shorter hospital stays, higher case turnover, coding optimization, and potential avoidance of financially unfavorable patients. These findings have direct policy relevance. One potential policy response may be to link reimbursement more closely to ethically relevant quality metrics, such as continuity of care, avoidable readmissions, and post-discharge deterioration, rather than coded activity alone [17, 18].

Managerial stakeholders likewise supported reforms aimed at reducing purely volume-driven incentives, although with different emphasis. Both groups expressed concern regarding increasing commercialization of hospital care, including stronger nonprofit governance structures and partial decoupling of hospital financing from case-based revenue alone [19–21]. Taken together, these findings are compatible with the concept of a hybrid financing model combining DRG-based reimbursement with protected structural or standby financing (“Vorhaltefinanzierung”) for high-acuity and continuity-intensive services.

This is directly relevant to the ongoing German hospital reform process, which seeks to reduce dependence on DRG-based payment in favor of greater structural financing [22]. Our results suggest that the central reform question is not only how much DRG influence should be reduced, but also where explicit ethical safeguards should be introduced [23]. Two mechanisms emerge from the data:

1. Quality-linked financing, connecting reimbursement to independently verifiable continuity and quality indicators for vulnerable or complex patients.
2. Structural or standby financing (“Vorhaltefinanzierung”), ensuring stable funding for clinically necessary but economically disadvantaged services such as intensive care, multimorbidity management, and complex internal medicine.

These mechanisms may help address the most frequently reported concerns in our sample: fragmentation of care and pressure to prioritize financially attractive patients. They also respond to concerns regarding “gaming” behaviors such as strategic coding and DRG optimization.

The findings should nevertheless be interpreted cautiously. The cross-sectional design captures perceptions at a single time point within a changing policy environment and does not permit causal inference. We cannot determine whether DRG exposure itself contributes to moral distress or whether individuals with particular professional value orientations perceive DRGs more critically. In addition, local staffing conditions, leadership style, and institutional climate may substantially influence these perceptions.

In broader health-system terms, the findings raise a central normative question: can hospital payment systems be aligned with professional obligations toward vulnerable and high-need patients without undermining economic sustainability? Our interpretation is that such alignment is possible, but requires deliberate integration of activity-based reimbursement with structural financing and quality safeguards. It is also important to recognize that the former per diem reimbursement system created its own economic incentives, particularly toward prolonged hospitalization. DRGs may therefore have shifted rather than created economic pressures within hospital care.

### Alternative reimbursement systems

The present findings also support the broader literature on alternative hospital payment systems. Several authors have argued that purely DRG-based reimbursement should be complemented by quality-linked financing, bundled payments, global budgets, or mixed models combining activity-based and structural funding. In the current German reform debate, the concept of standby or structural capacity financing aims to ensure that hospitals maintain clinically necessary but financially resource-intensive services such as intensive care, multimorbidity management, and emergency care. Our results suggest that a hybrid model combining DRGs with quality indicators and structural funding may reduce avoidable ethical tension, even though some degree of conflict is unavoidable whenever resources are finite. Ethical tension cannot be eliminated entirely in any health system characterized by finite resources. The aim of reimbursement reform should therefore be to reduce avoidable conflict and to support clinicians in balancing efficiency with professional obligations. Several alternative payment systems, including bundled payments, value-based care, and blended reimbursement models, have been proposed to align financial incentives with continuity, equity, and quality of care [17, 20].

### Practical implications

Several practical implications emerge from these findings. First, hospitals may benefit from stronger institutional support for ethical reflection, particularly in departments exposed to high economic pressure. Second, communication of financial targets by hospital leadership may influence whether economic constraints are experienced as compatible with professional ethics. Third, quality indicators emphasizing continuity of care, readmission avoidance, and vulnerable patient populations may help counterbalance purely throughput-oriented incentives.

### Limitations

#### Several limitations must be considered

First, the cross-sectional design allows only the identification of associations and perceptions; no causal inference can be made regarding whether DRG incentives directly produce moral distress. Second, the response rate of 19% raises the possibility of self-selection and non-response bias, although this range is comparable to many physician surveys. Respondents with particularly strong views regarding the DRG system may have been more likely to participate. However, this response rate is comparable to many surveys among hospital physicians and managers. Third, ethical tension may arise not only from DRG incentives but also from the broader reality of scarce resources and increasing demands on hospital systems. Therefore, our findings should not be interpreted as evidence that DRG-based reimbursement is inherently unethical or directly causes reduced quality of care. Rather, they reflect perceived ethical pressure under DRG conditions, which may also be shaped by staffing constraints, leadership culture, regional structures, and broader resource scarcity. Fourth, although the survey included both physicians and managerial stakeholders, other professional groups were either underrepresented or excluded from subgroup analysis in the final models. This limits the ability to characterize the full ethical ecosystem of hospital care delivery. Fifth, all measures are self-reported. Social desirability bias cannot be ruled out, especially in domains touching on “gaming,” early discharge, or risk selection. We did not independently verify reported practices against administrative data. Sixth, while missingness in key variables was low (each < 5%), we relied on pairwise deletion and complete-case analysis. This may introduce minor analytic bias, especially in subgroup comparisons. The ethical tensions observed in this study may not arise exclusively from DRG reimbursement but also from the broader reality of scarce resources in modern hospital systems. Because most respondents had not worked under the pre-2003 reimbursement system, findings reflect current perceptions of DRG-related incentives rather than direct comparisons between pre-DRG and post-DRG condition. The wording of some survey items may have emphasized economic pressure associated with DRGs and could therefore have influenced responses. Although the questionnaire was pilot-tested, framing effects and acquiescence bias cannot be excluded. The predominance of male respondents reflects the current gender distribution in senior hospital leadership positions in Germany and may limit generalizability to more gender-balanced healthcare settings.

## Conclusion

This study demonstrates that physicians and managerial stakeholders perceive DRG-linked hospital financing differently, particularly regarding professional autonomy, patient-centeredness, and moral conflict. These findings do not establish causal effects of DRG reimbursement itself but suggest that financing structures interact with organizational culture and professional role expectations in ethically relevant ways. Future hospital reforms may therefore benefit from integrating efficiency incentives with safeguards supporting continuity of care, professional integrity, and the treatment of vulnerable patient populations.

## Supplementary Information

Supplementary Material 1.

## Data Availability

The datasets generated and analyzed during the current study are not publicly available due to data protection regulations but are available from the corresponding author on reasonable request.

## Abbreviations

AI: Artificial intelligence
CI: Confidence interval
DRG: Diagnosis-related group
OR: Odds ratio
SD: Standard deviation
UHC: Universal health coverage
SPSS: Statistical Package for the Social Sciences
Stata: Statistical Data Analysis
