# Supplementary material for: Ethical perceptions of DRG-based hospital financing among physicians and hospital managers in Germany: a cross-sectional survey
Source: BMC Med Ethics. 2026 Jun 3;27:104. doi: 10.1186/s12910-026-01498-0 (PMC13231608; doi:10.1186/s12910-026-01498-0)
Supplement: Supplementary file 1 — Supplementary Material 1. [file 12910_2026_1498_MOESM1_ESM.docx]

**Supplement S1. Codebook**

**Overview**

This codebook provides detailed documentation of all variables included in the cross-sectional survey on *Ethical Perceptions of DRG-Based Hospital Financing in Germany*. Each variable entry specifies the original questionnaire item, response format, label, value range, missing-value code, and any data transformations performed prior to analysis (e.g., index construction, dichotomization, or recoding for logistic regression). Unless otherwise specified, all attitudinal survey items used a six-point Likert scale ranging from 1 = strongly disagree to 6 = strongly agree. No neutral midpoint was provided in order to encourage directional responses. All attitudinal survey items were measured using six-point Likert scales and treated primarily as ordinal variables. For regression analyses, selected variables were dichotomized into agreement versus disagreement categories as specified below. Missing values were coded as 99 and excluded using pairwise deletion for descriptive analyses and complete-case analysis for regression models.

**Section 1. Demographics and Professional Characteristics**

| Variable Name | Label / Description | Response Format / Coding | Value Range / Categories | Missing Code | Transformation / Derived Variable |
| --- | --- | --- | --- | --- | --- |
| id | Respondent ID (anonymized) | Numeric (auto-generated) | 1–529 | — | Unique identifier (no analysis use) |
| gender | Gender | Categorical | 1 = Male, 2 = Female, 3 = Other | 99 | — |
| age | Age (years) | Numeric (open entry) | 24–68 | 99 | Used as continuous; grouped into <40, 40–54, ≥55 for sensitivity analysis |
| profession | Professional group | Categorical | 1 = Physician, 2 = managerial stakeholders, 3 = Other | 99 | Recoded as prof_bin: 1 = Physician, 0 = managerial stakeholders (main model) |
| region | Federal state | Categorical | 1 = NRW, 2 = Saxony-Anhalt, | 99 | Used for subgroup analysis (NRW vs Saxony-Anhalt as reference) |
| facility | Facility type | Categorical | 1 = University hospital, 2 = General hospital, 3 = Rehabilitation clinic, 4 = Private practice | 99 | — |
| drg_experience | Direct DRG experience | Dichotomous | 1 = Yes, 0 = No | 99 | Used as main exposure variable |

**Section 2. Ethical Perception Variables**

| Variable Name | Survey Item (English translation) | Response Format / Coding | Value Range | Missing Code | Transformation / Derived Variable |
| --- | --- | --- | --- | --- | --- |
| eth_equity | “DRG incentives undermine equity in patient care.” | 6-point Likert | 1 = Strongly disagree → 6 = Strongly agree | 99 | Used descriptively as ordinal data and dichotomized for regression analyses where indicated; additionally included in exploratory composite ethical concern indices. eth_index |
| eth_autonomy | “DRGs restrict clinical autonomy in treatment decisions.” | 6-point Likert | 1–6 | 99 | — |
| eth_transparency | “The DRG system improves transparency in hospital financing.” | 6-point Likert (reverse-coded) | 1–6 | 99 | Recoded (R) so higher = more concern |
| eth_gaming | “Coding optimization (‘gaming’) is a widespread problem.” | 6-point Likert | 1–6 | 99 | — |
| eth_vulnerable | “Vulnerable patients (elderly, multimorbid) are disadvantaged under DRGs.” | 6-point Likert | 1–6 | 99 | — |
| eth_index | Composite ethical concern index | Continuous | Mean of eth_equity, eth_autonomy, eth_gaming, eth_vulnerable | — | Cronbach’s α = 0.84 |

**Section 3. Perceived Organizational Effects**

| Variable Name | Survey Item | Response Format / Coding | Value Range | Missing Code | Transformation / Derived Variable |
| --- | --- | --- | --- | --- | --- |
| time_pressure | “Economic pressures have increased time constraints in patient care.” | 6-point Likert | 1–6 | 99 | — |
| early_discharge | “DRGs incentivize premature discharge.” | 6-point Likert | 1–6 | 99 | — |
| continuity_loss | “Continuity of care has declined under DRG conditions.” | 6-point Likert | 1–6 | 99 | — |
| upcoding_awareness | “Upcoding is common in my department.” | 6-point Likert | 1–6 | 99 | — |

**Section 4. Moral Distress and Ethical Climate**

| Variable Name | Survey Item | Response Format / Coding | Value Range | Missing Code | Transformation / Derived Variable |
| --- | --- | --- | --- | --- | --- |
| moral_distress | “I experience moral distress due to economic constraints.” | 6-point Likert | 1–6 | 99 | Dependent variable in logistic regression |
| ethical_support | “Ethical reflection is institutionally supported in my workplace.” | 6-point Likert | 1–6 | 99 | Reverse-coded (R) for interpretability |
| eth_climate_index | Composite ethical climate score | Composite ordinal summary score derived from Likert-scale items. | Mean of moral_distress (R), ethical_support, transparency (R) | — | Higher = more positive ethical climate |

**Section 5. Derived and Analytical Variables**

| Variable Name | Definition / Derivation Rule | Value Range / Type | Use in Models |
| --- | --- | --- | --- |
| md_high | Binary moral distress indicator (moral_distress ≥4) | 0 = Low/Moderate, 1 = High | Logistic regression dependent variable |
| equity_high | Equity concern (Likert ≥4) | 0 = Low, 1 = High | Subgroup regression |
| prof_bin | Profession binary recode (1 = Physician, 0 = managerial stakeholders) | 0–1 | Covariate in all main models |
| region_NRWSA | Regional comparison variable (1 = NRW, 0 = Saxony-Anhalt) | 0–1 | Regional logistic model |
| eth_index_std | Standardized composite ethical concern score (z-transformed) | Standardized exploratory composite score derived from ordinal Likert-scale items. | Used for correlation and regression |
| drg_exp_x_prof | Interaction term (DRG experience × profession) | Continuous | Tested in moderation analysis |

**Supplement S2. STROBE 22-Item Checklist for Cross-Sectional Studies**

| Item No. | Section | STROBE Recommendation (Condensed) | Location in Manuscript |
| --- | --- | --- | --- |
| 1 | **Title and Abstract** | Indicate the study design with a commonly used term in the title or abstract and provide an informative, balanced summary. | Title; Abstract |
| 2 | **Background/Rationale** | Explain the scientific background and rationale for the investigation. | Introduction, paragraph 1–2 |
| 3 | **Objectives** | State specific objectives, including any prespecified hypotheses. | Introduction, last paragraph |
| 4 | **Study Design** | Present key elements of the study design early in the paper. | Methods, first paragraph |
| 5 | **Setting** | Describe the setting, locations, and relevant dates, including periods of recruitment and data collection. | Methods → Setting and Participants |
| 6 | **Participants** | Give eligibility criteria, sources and methods of participant selection, and methods of follow-up if applicable. Include a flow diagram. | Methods → Participants; Figure 2 (Flow of participants) |
| 7 | **Variables** | Clearly define all outcomes, exposures, predictors, potential confounders, and effect modifiers. Give diagnostic criteria if applicable. | Methods → Variables and Definitions |
| 8 | **Data Sources/Measurements** | For each variable of interest, give data sources and details of assessment (measurement). | Methods → Survey Instrument; Supplement Table S1 |
| 9 | **Bias** | Describe efforts to address potential sources of bias (e.g., non-response bias, recall bias). | Methods → Limitations subsection |
| 10 | **Study Size** | Explain how the study size was determined. | Methods → Sampling and Recruitment |
| 11 | **Quantitative Variables** | Explain how quantitative variables were handled in the analyses; describe groupings if applicable. | Methods → Statistical Analysis |
| 12 | **Statistical Methods** | Describe all statistical methods, including control for confounding, subgroup analyses, and missing data handling. | Methods → Statistical Analysis |
| 13 | **Participants (Results)** | Report the numbers of individuals at each stage of the study—e.g., eligible, examined, included—and reasons for non-participation. | Results → Participant Characteristics; Figure 1 |
| 14 | **Descriptive Data** | Provide characteristics of study participants and information on exposures and potential confounders. Indicate number of participants with missing data for each variable. | Results → Table 1; Supplement Table S1 |
| 15 | **Outcome Data** | Report numbers of outcome events or summary measures. | Results → Main Findings |
| 16 | **Main Results** | Provide unadjusted estimates and, if applicable, adjusted estimates and their precision (e.g., 95% CI). Clarify confounders included in adjustments. | Results → Tables 2–4 |
| 17 | **Other Analyses** | Report subgroup, interaction, and sensitivity analyses. | Results → Subgroup Analyses; Supplement S3 |
| 18 | **Key Results** | Summarize key results with reference to study objectives. | Discussion → Paragraph 1 |
| 19 | **Limitations** | Discuss limitations of the study, considering sources of bias or imprecision. Discuss direction and magnitude of potential bias. | Discussion → Limitations |
| 20 | **Interpretation** | Provide a cautious interpretation of results, considering objectives, limitations, and other relevant evidence. | Discussion → Interpretation |
| 21 | **Generalisability** | Discuss the external validity (generalisability) of the study results. | Discussion → Implications and Transferability |
| 22 | **Funding and Ethical Approval** | Give source of funding and role of funders; describe ethical approval and consent procedures. | End-Matter → Funding, Ethics, COI, Data Availability |

**Supplement S3. Data Transparency and Reproducibility Statement**

Aggregated and fully de-identified survey data, along with the complete analysis scripts (SPSS, Stata, and R versions), are available from the corresponding author upon reasonable academic request. Data sharing complies with the study’s ethics approval and the German Federal Data Protection Act (BDSG).

To protect respondent anonymity, no individual-level or institution-identifying information can be released. Shared materials include:

- Variable-level aggregated data tables and codebook entries;
- Annotated analysis syntax reproducing descriptive statistics, regression models, and figures;
- The STROBE checklist and supplementary documentation (Questionnaire, Codebook, Analytic Pipeline).

Requests should be directed to the corresponding author and will be considered for non-commercial academic research consistent with the study’s ethical approval and data-use agreement.

All analyses were performed using R version 4.3.2 (R Foundation for Statistical Computing, Vienna, Austria), SPSS v29.0 (IBM Corp., Armonk, NY), and Stata v18.0 (StataCorp LLC, College Station, TX) to ensure reproducibility across platforms.

**Supplement S3-Questionnaire**

# Diagnosis-Related Groups (DRGs) in the Context of Medical Ethics

Dear participant,

With the introduction of diagnosis-related groups (DRGs), hospitals have increasingly oriented their operations toward economic goals. Whereas resources were once allocated by daily and nursing rates, under the DRG system the services defined for each diagnosis are reimbursed with a fixed amount. Since the introduction of diagnosis-related groups (DRGs), hospital reimbursement in Germany has increasingly been based on fixed payments per case rather than per diem rates. This system has changed how hospitals organize care and allocate resources. The present survey explores how physicians and managerial stakeholders perceive these changes. The introduction of DRGs aimed to remove misaligned incentives, increase efficiency in hospital service delivery, and reduce costs.

Against this background, we (Sara Bagherzadeh, Prof. Dr. med. Michael Buerke, and Dr. Priyanka Boettger) initiated the scientific project “Diagnosis-Related Groups in the Context of Medical Ethics” to examine the effects of DRGs on hospital practice and ethical aspects. The study investigates how physicians and managerial stakeholders perceive changes in medical and ethical conduct after DRG implementation in two German regions: Saxony-Anhalt (East) and North Rhine-Westphalia (West).

Your participation is very important to us. All responses will be treated confidentially and will not be shared. Completing the questionnaire will take no more than ten minutes. You will, of course, be informed of the study results by e-mail.

michael.buerke@medizin.uni-halle.de
[bagherzadeh.sara@yahoo.com](mailto:bagherzadeh.sara@yahoo.com) Priyanka.boettger@uni-giessen.de

## 1. Motivation and Qualification

1.1 I am satisfied with my work.

☐ Strongly disagree ☐ Disagree ☐ Slightly disagree ☐ Slightly agree ☐ Agree ☐ Strongly agree

1.2 I am dissatisfied with my working environment.

☐ Strongly disagree ☐ Disagree ☐ Slightly disagree ☐ Slightly agree ☐ Agree ☐ Strongly agree

1.3 I have the following scope of action and decision-making authority in our hospital (mark one per row):

| Item | Strongly disagree | Disagree | Somewhat disagree | Somewhat agree | Agree | Strongly agree |
| --- | --- | --- | --- | --- | --- | --- |
| Leadership responsibility | ☐ | ☐ | ☐ | ☐ | ☐ | ☐ |
| Budget responsibility | ☐ | ☐ | ☐ | ☐ | ☐ | ☐ |
| Authority to issue instructions | ☐ | ☐ | ☐ | ☐ | ☐ | ☐ |
| Decision-making competence | ☐ | ☐ | ☐ | ☐ | ☐ | ☐ |
| Personnel competence | ☐ | ☐ | ☐ | ☐ | ☐ | ☐ |

1.4 The following tasks fall within my area of responsibility (check all that apply):

| Item | Strongly disagree | Disagree | Somewhat disagree | Somewhat agree | Agree | Strongly agree |
| --- | --- | --- | --- | --- | --- | --- |
| Employee supervision and appraisal | ☐ | ☐ | ☐ | ☐ | ☐ | ☐ |
| Personnel and budget management | ☐ | ☐ | ☐ | ☐ | ☐ | ☐ |
| Staff development according to service portfolio planning | ☐ | ☐ | ☐ | ☐ | ☐ | ☐ |
| Quality management | ☐ | ☐ | ☐ | ☐ | ☐ | ☐ |
| Hygiene management | ☐ | ☐ | ☐ | ☐ | ☐ | ☐ |
| DRG controlling | ☐ | ☐ | ☐ | ☐ | ☐ | ☐ |

1.5 The introduction of DRGs has reduced physicians’ enjoyment of their work.

☐ Strongly disagree ☐ Disagree ☐ Slightly disagree ☐ Slightly agree ☐ Agree ☐ Strongly agree

## 2. Sociodemographic Factors

2.1 Age (years) ☐ <30 ☐ 30–40 ☐ 41–50 ☐ >50

2.2 Gender ☐ Male ☐ Female

2.3 Professional experience (years) ☐ <5 ☐ 5–10 ☐ 11–15 ☐ >15

2.4 Position ☐ Chief physician ☐ Senior physician ☐ Hospital management ☐ Controlling management ☐ Human resources management

2.5 Years in current position ☐ <5 ☐ 5–10 ☐ 11–15 ☐ >15

2.6 Hospital size ☐ Primary/basic care ☐ Specialist care ☐ Maximum care

2.7 Hospital ownership ☐ Public ☐ Non-profit ☐ Private

## 3. External Factors Influencing Medical Decisions (Individual Level)

3.1 The self-perception of physicians and managerial stakeholders differs fundamentally.

☐ Strongly disagree ☐ Disagree ☐ Slightly disagree ☐ Slightly agree ☐ Agree ☐ Strongly agree

3.2 Medical decisions are increasingly influenced by economic profitability considerations.

☐ Strongly disagree ☐ Disagree ☐ Slightly disagree ☐ Slightly agree ☐ Agree ☐ Strongly agree

3.3 A physician in a leadership position must also see themselves as a manager.

☐ Strongly disagree ☐ Disagree ☐ Slightly disagree ☐ Slightly agree ☐ Agree ☐ Strongly agree

3.4 Economic considerations may reduce the quality of patient care.

☐ Strongly disagree ☐ Disagree ☐ Slightly disagree ☐ Slightly agree ☐ Agree ☐ Strongly agree

## 4. External Factors Influencing Medical Decisions (Hospital Level)

4.1 Economic considerations take priority in health-care delivery.

☐ Strongly disagree ☐ Disagree ☐ Slightly disagree ☐ Slightly agree ☐ Agree ☐ Strongly agree

4.2 Financial incentives divert attention from patient-oriented care.

☐ Strongly disagree ☐ Disagree ☐ Slightly disagree ☐ Slightly agree ☐ Agree ☐ Strongly agree

4.3 The DRG system supports the selection of patients whose DRGs are profitable.

☐ Strongly disagree ☐ Disagree ☐ Slightly disagree ☐ Slightly agree ☐ Agree ☐ Strongly agree

4.4 In my hospital, lucrative patients are given preference.

☐ Strongly disagree ☐ Disagree ☐ Slightly disagree ☐ Slightly agree ☐ Agree ☐ Strongly agree

4.5 Since the introduction of DRGs, the following shifts have occurred (check all that apply):

☐ From unconditional help → profitability of performance
☐ From a relationship of trust → a contractual relationship
☐ From unquestioned caregiving → accountability for every action
☐ From compassionate practice → market-oriented service
☐ None

## 5. Internal Factors Influencing Medical Decisions (Individual Level)

5.1 Every patient can be treated with appropriate care.

☐ Strongly disagree ☐ Disagree ☐ Slightly disagree ☐ Slightly agree ☐ Agree ☐ Strongly agree

5.2 There is too little time for communication with patients.

☐ Strongly disagree ☐ Disagree ☐ Slightly disagree ☐ Slightly agree ☐ Agree ☐ Strongly agree

5.3 Compassion and attentiveness are increasingly sacrificed in favor of economic efficiency.

☐ Strongly disagree ☐ Disagree ☐ Slightly disagree ☐ Slightly agree ☐ Agree ☐ Strongly agree

5.4 A “revolving-door effect” is increasingly observed, where patients are readmitted soon after early discharge in an unstable condition.

☐ Strongly disagree ☐ Disagree ☐ Slightly disagree ☐ Slightly agree ☐ Agree ☐ Strongly agree

5.5 Which criteria influence decisions regarding discharge timing? (check all that apply):

☐ Individual clinical judgment
☐ Diagnosis-specific scope of services
☐ Efficiency of the treatment process
☐ Clinical outcomes
☐ Patient’s social environment
☐ DRG reimbursement incentive
☐ Type of procedure
☐ Patient’s general health status (morbidity, age, psychological state, mobility)
☐ Complete recovery
☐ Potential for further diagnostic evaluation (patient profitability)
☐ Bed shortages on the ward
☐ Length-of-stay limits (upper and lower thresholds)
☐ None

5.6 Since the introduction of DRGs, the trend toward fragmentation of treatments (case splitting and rising case numbers) has increased.

☐ Strongly disagree ☐ Disagree ☐ Slightly disagree ☐ Slightly agree ☐ Agree ☐ Strongly agree

5.7 Since the introduction of DRGs, patients are assigned more diagnoses.

☐ Strongly disagree ☐ Disagree ☐ Slightly disagree ☐ Slightly agree ☐ Agree ☐ Strongly agree

5.8 Since the introduction of DRGs, more procedures are performed that are not strictly necessary.

☐ Strongly disagree ☐ Disagree ☐ Slightly disagree ☐ Slightly agree ☐ Agree ☐ Strongly agree

5.9 The DRG system creates perverse incentives that are not always in the patient’s best interest.

☐ Strongly disagree ☐ Disagree ☐ Slightly disagree ☐ Slightly agree ☐ Agree ☐ Strongly agree

5.10 Since the introduction of DRGs, the quality of care has changed as follows (mark one per row):

| Aspect of care | Excellent | Very good | Good | Poor | Very poor | Miserable |
| --- | --- | --- | --- | --- | --- | --- |
| Accessibility of care | ☐ | ☐ | ☐ | ☐ | ☐ | ☐ |
| Appropriateness of care | ☐ | ☐ | ☐ | ☐ | ☐ | ☐ |
| Coordination of care | ☐ | ☐ | ☐ | ☐ | ☐ | ☐ |
| Economic efficiency of care | ☐ | ☐ | ☐ | ☐ | ☐ | ☐ |
| Patient orientation of care | ☐ | ☐ | ☐ | ☐ | ☐ | ☐ |
| Timeliness of care | ☐ | ☐ | ☐ | ☐ | ☐ | ☐ |

5.11 There is a conflict between the daily work of providing the best possible patient care and ensuring economic efficiency for the hospital.

☐ Strongly disagree ☐ Disagree ☐ Slightly disagree ☐ Slightly agree ☐ Agree ☐ Strongly agree

5.12 For me, optimal patient care includes (check all that apply):

| Item | Strongly disagree | Disagree | Somewhat disagree | Somewhat agree | Agree | Strongly agree |
| --- | --- | --- | --- | --- | --- | --- |
| Freedom of therapeutic decisions from financial considerations | ☐ | ☐ | ☐ | ☐ | ☐ | ☐ |
| Sufficient time with the patient | ☐ | ☐ | ☐ | ☐ | ☐ | ☐ |
| Individualised indication setting | ☐ | ☐ | ☐ | ☐ | ☐ | ☐ |
| Second medical opinion | ☐ | ☐ | ☐ | ☐ | ☐ | ☐ |
| Discussion of treatment needs and necessities | ☐ | ☐ | ☐ | ☐ | ☐ | ☐ |
| Rapid provision of care | ☐ | ☐ | ☐ | ☐ | ☐ | ☐ |
| Rationing of care | ☐ | ☐ | ☐ | ☐ | ☐ | ☐ |

## 6. The Health-Care System as a Market

6.1 Since the introduction of DRGs, the hospital’s caregiving role has transformed into a market-oriented hospital system.

☐ Strongly disagree ☐ Disagree ☐ Slightly disagree ☐ Slightly agree ☐ Agree ☐ Strongly agree

6.2 Our hospital generates a profit.

☐ Strongly disagree ☐ Disagree ☐ Slightly disagree ☐ Slightly agree ☐ Agree ☐ Strongly agree

6.3 The patient is now perceived as a customer within the health-care system.

☐ Strongly disagree ☐ Disagree ☐ Slightly disagree ☐ Slightly agree ☐ Agree ☐ Strongly agree

6.4 Patients now see themselves as customers with clearly defined expectations since the introduction of DRGs.

☐ Strongly disagree ☐ Disagree ☐ Slightly disagree ☐ Slightly agree ☐ Agree ☐ Strongly agree

6.5 The DRG system transforms the patient into an economic subject.

☐ Strongly disagree ☐ Disagree ☐ Slightly disagree ☐ Slightly agree ☐ Agree ☐ Strongly agree

6.6 The DRG system shifts the patient–physician relationship toward a provider–customer relationship.

☐ Strongly disagree ☐ Disagree ☐ Slightly disagree ☐ Slightly agree ☐ Agree ☐ Strongly agree

6.7 Hospital controlling aims to improve quality and efficiency through (check all that apply):

☐ Target agreements
☐ Bonus payments to senior physicians
☐ Special contractual clauses
☐ Certification
☐ Quality initiatives
☐ Benchmarking
☐ Controlling reports
☐ Standard Operating Procedures (SOPs)
☐ Combined quality and efficiency programs
☐ None

6.8 The introduction of DRGs—and partly competition between hospitals—has reduced treatment costs without compromising quality.

☐ Strongly disagree ☐ Disagree ☐ Slightly disagree ☐ Slightly agree ☐ Agree ☐ Strongly agree

## 7. Ethics and Values

7.1 There is a conflict between medical ethics and a hospital’s economic interests.

☐ Strongly disagree ☐ Disagree ☐ Slightly disagree ☐ Slightly agree ☐ Agree ☐ Strongly agree

7.2 Since the introduction of DRGs, my ethical attitude toward patients may have been affected by economic pressure.

☐ Strongly disagree ☐ Disagree ☐ Slightly disagree ☐ Slightly agree ☐ Agree ☐ Strongly agree

7.3 Benchmarking in controlling (analyzing deviations from targets as success/failure) is ethically justifiable (mark one per row):

| Aspect | Strongly disagree | Disagree | Slightly disagree | Slightly agree | Agree | Strongly agree |
| --- | --- | --- | --- | --- | --- | --- |
| Economic aspects | ☐ | ☐ | ☐ | ☐ | ☐ | ☐ |
| Qualitative aspects | ☐ | ☐ | ☐ | ☐ | ☐ | ☐ |

7.4 The DRG system obliges physicians not only to act for the patient’s welfare but also explicitly to practice cost-efficient medicine.

☐ Strongly disagree ☐ Disagree ☐ Slightly disagree ☐ Slightly agree ☐ Agree ☐ Strongly agree

7.5 Financial orientation in health care is ethically questionable.

☐ Strongly disagree ☐ Disagree ☐ Slightly disagree ☐ Slightly agree ☐ Agree ☐ Strongly agree

7.6 Rationing (withholding measures for cost reasons) is ethically acceptable within the DRG system.

☐ Strongly disagree ☐ Disagree ☐ Slightly disagree ☐ Slightly agree ☐ Agree ☐ Strongly agree

7.7 Moral or ethical conflicts are experienced in balancing quality, cost-efficiency, and fair access.

☐ Strongly disagree ☐ Disagree ☐ Slightly disagree ☐ Slightly agree ☐ Agree ☐ Strongly agree

7.8 There is a need to strengthen non-profit-oriented medicine in hospitals.

☐ Strongly disagree ☐ Disagree ☐ Slightly disagree ☐ Slightly agree ☐ Agree ☐ Strongly agree

Comments / Ideas / Suggestions:
____________________________________
____________________________________

**Thank you for your participation!**

Sara Bagherzadeh Prof. Dr. med. Michael Buerke Dr. Priyanka Boettger

**Supplement Table S4.** Multivariable logistic regression analyses of perceived ethical tensions associated with DRG-based hospital financing

| Outcome Variable | Predictor | Odds Ratio (OR) | 95% CI | p-value |
| --- | --- | --- | --- | --- |
| Moral conflict due to DRGs | Physician role (vs managerial stakeholder) | 2.75 | 1.60–4.72 | 0.001 |
| Reduced patient-centeredness | Physician role (vs managerial stakeholder) | 1.89 | 1.12–3.19 | 0.018 |
| Reduced patient-centeredness | Private ownership (vs public) | 1.48 | 1.02–2.13 | 0.039 |
| Perceived fragmentation of care | NRW vs Saxony-Anhalt | 1.36 | 0.95–1.93 | 0.092 |

*Models adjusted for years of professional experience, hospital type (primary/specialized/university), ownership structure (public/non-profit/private), and region. Missing values were handled using complete-case analysis. Interaction terms were explored but did not materially alter the main findings. No evidence of problematic multicollinearity was observed.*
